# A content analysis of 2023 Türkiye general election pledges on public health nutrition and related sustainable development goals

**DOI:** 10.1186/s41043-026-01299-6

**Published:** 2026-04-26

**Authors:** Esra Nur Güner, Gönenç Uysal, Zeynep Begüm Kalyoncu Atasoy

**Affiliations:** 1 Graduate School of Health Sciences, Department of Global Health, Koç University, Rumelifeneri, Sarıyer, 34450 Istanbul Türkiye; 2 School of Global Affairs, Lancaster University, Lancaster, LA1 4YW UK; 3 Faculty of Health Sciences, Department of Nutrition and Dietetics, University of Health Sciences Gülhane, 06018 Ankara, Türkiye

**Keywords:** Nutrition policy, Election declarations, Food security, Qualitative research, Türkiye

## Abstract

**Background:**

Giving priority to public health nutrition policies can improve health quality and national development. An intersecting analysis of public health nutrition (PHN) and political pledges is limited globally, and in the context of Türkiye.

**Methods:**

This study aimed to analyze the emphasis given by political parties and alliances to policies related to public health nutrition (PHN) in their publicly available documents and to identify whether their proposals reflect a broad understanding of nutrition-related public health issues. This study employed a qualitative research design utilizing the content analysis method. Publicly available pledges including election declarations, party programs, and formal website-based documents were analyzed in the study. The website-based documents were analyzed through qualitative content analysis via MAXQDA (Version 2022.8) software. This study consisted of two distinct analyses. The first analysis was conducted through themes of nutrition security, food supply, food safety, sustainable agriculture, and cultural sensitivity. In the second analysis, documents were analyzed in relation to specific four Sustainable Development Goals (SDGs) namely Goal 2 (Zero Hunger), Goal 12 (Responsible Consumption and Production), Goal 5 (Gender Equality), and Goal 6 (Clean Water and Sanitation), all of which are relevant to PHN.

**Results:**

Overall, the documents were coded 1209 times in the first analysis on PHN, while were coded 941 times in the second analysis on SDGs. The main opposition Nation Alliance (NA) had the most coded documents followed by the ruling People’s Alliance (PA). After sustainable agriculture, the majority of policies were produced in the theme of food security, followed by food safety, food supply, and cultural sensitivity respectively. For SDGs, Goal 2 (Zero Hunger) was the most coded theme followed by Goal 12 (Responsible Consumption and Production), Goal 5 (Gender Equality), and Goal 6 (Clean Water and Sanitation) respectively. The category of sustainable nutrition has not been used in the documents of any party or alliance. Moreover, the dietitian profession legislation has been proposed by only two alliances.

**Discussion:**

Parties/alliances remained inadequate in offering pledges in relation to PHN and related SDG goals.

**Conclusions:**

The findings provide evidence-based resources to emphasize this area, raising awareness among researchers, policy advocates, and policymakers, and ultimately contributing to the development of sustainable, accountable, and country-specific policies.

**Supplementary Information:**

The online version contains supplementary material available at 10.1186/s41043-026-01299-6.

## Background

In recent years, shifts in dietary patterns driven by multifaceted challenges have significantly increased the prevalence and incidence of nutrition-related diseases [1]. The World Obesity Atlas 2023 projects that the yearly worldwide economic cost of overweight and obesity will reach $4.32 trillion by 2035 if preventive and treatment strategies do not improve. This atlas projects the economic impact of overweight to reach $35,846 million annually by 2035 in Türkiye [2]. This shows the necessity of policies addressing public health nutrition problems to create a healthy population and support national development.

Nutrition policies aim to promote health and prevent nutrition-related deficiencies and diseases. The general framework of nutrition-related policies includes setting standards and guidelines, making healthy nutrition accessible to the entire society, providing education and raising awareness, labeling, food fortification, and promoting, restricting, or prohibiting unhealthy foods or food components [3]. Previous research has highlighted the critical interplay between policy decisions and public health outcomes [4]. Across the globe, many countries have introduced nutrition-related policies; for instance, Mexico’s nationwide tax reform on sugar-sweetened beverages and caloric-dense foods exempting basic staples such as sugar, bread, and rice resulted in decreased purchasing [5]. Subsidies on healthier foods, launched in the USA, Canada, France, Germany, Netherlands, South Africa, and the UK, increased the sale of these foods [6]. Food fortification with folic acid policy in 60 countries prevents most cases of neural tube defects [7] As part of these global efforts, in Türkiye, various public health initiatives have shown significant impact. The “Türkiye Salt Reduction Program,” launched in 2011, successfully reduced salt consumption through public campaigns and regulations according to the Türkiye Nutrition and Health Survey (TNHS) [8–10]. The “Action Plan for Prevention and Control of Obesity and Physical Activity” (2019–2023) and “The School Milk Project” also contributed to improved nutrition [11, 12]. Additionally, the “Iron-Like Türkiye” project (2004) decreased iron deficiency anemia in babies by providing free iron supplements. These examples highlight the effectiveness of targeted health policies [13].

Nevertheless, PHN in Türkiye has faced various economic, demographic, and environmental challenges, especially affecting vulnerable groups such as children, disabled people, and natural disaster survivors. Along with rising food inflation, the declining consumer power has made it difficult to eat healthily [14]. Combined with mass and uncontrolled migration, the rapidly growing population is generating a need for increased food supply, and failure to meet this need is driving food inflation in Türkiye [15–17]. Furthermore, in recent years, food contamination, especially in workplaces, schools, and student dormitories, has resulted in an increase in food-borne illness in Türkiye that makes the food safety a crucial problem. The price increases in cafeterias have affected the food security of workers and students, further resulting in protests [18–20]. Moreover, one would expect that public nutrition has been safeguarded during and after critical situations such as natural disasters. However, the Türkiye-Syria earthquake, which was a devastating earthquake that occurred on 6 February 2023, deepened food safety and security problems [21] According to the report of The Union of Chambers of Turkish Engineers and Architects (UCTEA), hot food distribution started only after the fourth day of the earthquake and the nutritional needs of the earthquake survivors in the priority group could only be partially met after the sixth day. Cold supply chain and transportation and storage vehicles could not reach many places, and damage assessment studies and food production in agriculture and livestock could not be conducted [22]. Climate crisis is the other significant factor affecting nutrition [23]. In Türkiye, the destructive impact of climate crisis can be observed in the recent floods in Sanliurfa and Rize by causing damage to agricultural lands [24, 25]. All these challenges make it essential to look at PHN through a comprehensive lens, including sustainable agriculture, nutrition, and food policies, so that the PHN can be improved.

The multi-party system in Türkiye compels politicians to use many strategies to get votes. These strategies include making policy promises that address the needs of various segments of society to secure votes. Considering the increasingly challenging living conditions in Türkiye, nutrition and food policies are expected to play a vital role in voters’ decision-making. In the context of PHN, election manifestos should reflect responses to socio-economic challenges, such as food inflation, food insecurity, declining purchasing power, and population growth. Therefore, it is crucial for policymakers to prioritize nutrition-related policies and also for political actors to shape their electoral promises accordingly. This motivates the need to analyze how political parties and alliances address existing issues in their election declarations. However, an intersecting analysis of PHN and political pledges is limited globally, and in the case of Türkiye. While, literature tends to evaluate agriculture, industry, and tourism policies in party programs, existing agriculture, and nutrition policies, there is a dearth of literature on the analyses of pledges in election declarations/party programs in the context of PHN policies [26–29]. We, therefore, aimed to fill this gap by analyzing the election pledges of various political parties and alliances participating in the 14 May 2023 Türkiye General Election -which will be stated as the 2023 Türkiye Election for the rest of the article- and shed light on how political actors approach PHN issues.

## Method

### Study design

Content analysis methodology is used to understand the characteristics, trends, and patterns of content by systematically examining text or media content. This qualitative research method assumes that texts are the facts to be examined and hence were used as units of data analysis [30]. Content analysis was preferred because it systematically reveals patterns in the texts and ensures objectivity and repeatability [31].

In this study, two analyses were conducted to see how election declarations or party programs address public health nutrition within both domestic and global contexts. The party programs, pledges, and election declarations prepared by the alliances/parties participating in the 2023 Türkiye Election and published on their official websites for public dissemination were examined for the domestic context of our study. On the other hand, for the global context, Sustainable Development Goals (SDGs) with direct relevance to nutrition were first identified among the global targets established by the United Nations (UN) to be achieved by 2030, and the documents were subsequently analyzed for their alignment with these goals [32].

Identical or substantially similar pledges were coded separately when they appeared in the official documents of different parties. Even when the content overlapped, no aggregation or de-duplication was performed; each pledge was treated as a distinct unit of analysis and counted according to the specific party or alliance document in which it appeared.

The present study was approved by the Istanbul Aydin University Non-Interventional Clinical Research Ethics Committee (Decision Number: B.30.2.AYD.0.00.00–050.06.00.06.04/126).

### Data sources and eligibility criteria

Since the study focused on selecting a specific set of declarations/party programs based on defined criteria, a purposive sampling method was used. This method involves the intentional selection of documents that meet the inclusion and exclusion criteria. Purposive sampling improves a study’s validity and the reliability of the data by selecting a sample that matches the research aims and objectives [33].

Documents were collected in September 2023. Documents retrieved for this study were available on the websites of members and supporters of the People’s Alliance (PA), Nation’s Alliance (NA), Ancestral Alliance (AA), the Union of Socialist Forces (USF) and Labor and Freedom Alliance (LFA) and non-aligned parties participated in the 2023 Türkiye Election (Table 1) [34]. The PA is under the leadership of the government party -Justice and Development Party (AKP, Adalet ve Kalkinma Partisi) whereas the main opposition is represented by the NA. Some parties did not participate in the alliances officially but declared their support unofficially. Therefore, these parties were regarded as “de facto alliances” in this study (Table 1). Members and supporters of alliances as well as parties participating independently in the election were included in the analysis. Parties that were not eligible to participate in the election, because they did not meet the legal and administrative requirements, and/or did not support any alliance were excluded from the study. In cases where no specific election declaration was announced, general information, pledges, and/or party programs on the parties’ websites were analyzed. The study examined only written sources, excluding discourses or visual sources. Forty-four political parties participated or supported alliances in the election without participating, and among them, a total of thirteen parties participated in the election without being included in any alliance (Supplementary Table 1**)**. For alliances that have joint policy statements, their party programs and declarations were also included alongside the joint policy statements. In other alliances without joint policy statements, only the documents of the non-aligned parties were included.

**Table 1:** Political parties and alliance membership with program presence in the 2023 türkiye general election* [34]

Alliance/party	Members	Presence of party programs/declarations
PA	AKP, MHP, HÜDAPAR, YRP, BBP, DSP	✔ (Joint policy statement does not exist)
NA	CHP, İYİP, SP, DP, DEVA, GP	✔ (Joint policy statement exists)
LFA	HDP, TİP, EMEP, YSP, EHP^x^, TÖP^x^	✔ (Joint policy statement exists)
AA	ZP, AP, HÜRVATAN^x, y^, GAAP^x, y^, ÜP^x^, TÜİP^x^	✔ (Joint policy statement does not exist)
USF	SOL, TKP, TKH, DİP^x, y^, DH^x^, SCP^x, y^, TSİP^x^	✔ (Joint policy statement does not exist)
MP^a^		✔
ANAP		**-**
VP		✔
BTP^a^		✔
GBP		**-**
MİLLİYOL		✔
HKP		✔
YP		✔
AB PARTY		✔
BTP^b^		**-**
HAK-PAR		✔
MP^b^		**-**
GENÇPARTİ		**-**

*Turkish abbreviations of the parties are given in Supplementary Table 1. ^x^Parties that are not eligible to participate in the election., ^y^De facto alliance, ^a, b^The parties having the same initials but having different names are also provided in Supplementary Table 1

### Data analysis

The data were organized, coded, and analyzed via MAXQDA (Version 2022.8) software, on the main themes related to PHN and targets of the UN SDGs directly related to PHN: Goal 2: Zero Hunger, Goal 5: Gender Equality, Goal 6: Clean Water and Sanitation, Goal 12: Responsible Consumption and Production [35].

The first analysis was regarding PHN and five overarching constructs were operationalized: (1) nutrition security (2) food supply (3) food safety (4) sustainable agriculture (5) cultural sensitivity based on a combination of inductive and deductive approach. During the coding process, themes emerged from a close reading of the documents (inductive approach), reflecting recurrent patterns and priorities in PHN. Simultaneously, the selection was informed by established conceptual frameworks in the literature, particularly dimensions of food security and nutrition security (deductive approach) [36]. Within the PHN framework, line-by-line coding enabled the emergence of additional themes and thus finalized the themes that were used in the analysis (Supplementary Table 2) [37]. The second analysis was regarding SDGs and only four constructs that have specific targets directly linked to PHN were operationalized (Supplementary Table 3), namely (1) Goal 2: Zero Hunger (2) Goal 5: Gender Equality (3) Goal 6: Clean Water and Sanitation (4) Goal 12: Responsible Consumption and Production (Supplementary Table 4).

The evaluation of PHN policies was approached from a strong social justice perspective. For the multidisciplinary team, consisting of two female public health nutritionists and a progressive female political scientist, current issues such as food inflation and food security, which are critical concerns for the Turkish public, were regarded as matters of utmost importance. The concepts of the right to food, health disparities, and food inequity were adopted as central principles, with a focus on how access to this right can be secured through policies that embrace social justice and sustainable agriculture. In this context, an analysis was conducted on how the parties and alliances approached these issues.

We followed the Consolidated Criteria for reporting qualitative research (COREQ) guideline while reporting this paper [38].

## Results

Qualitative analyses revealed that different parties/alliances exhibit distinct patterns **(**Supplementary Table 2). Generally, the alliances that produce the most policies in relevant areas are the NA and the PA. The main opposing alliance NA has emphasized food security, healthy nutrition, and food safety more than other alliances and parties. Food waste has been most prominently highlighted by the Homeland Party (MP^a^, Memleket Partisi), a non-aligned party with an opposing agenda to the ruling government. The pledge to provide free meals to ensure food security and healthy nutrition has been made by both the LFA and the NA that are both opposing alliances. Policies regarding nutrition in emergencies have been mostly formulated by the NA. The LFA has grounded food security in the context of food inflation and poverty in Türkiye. Overall, the AA and the USF have produced fewer policies with regard to PHN compared to other alliances. Among the parties participating in the election individually, the MP^a^ has made the most policies by far on the relevant topics. The second analysis revealed that the NA, PA, LFA, and MP^a^ have had the highest number of codes related to SDGs compared to other alliances and parties.

A. Public health nutrition

**Fig. 1:**
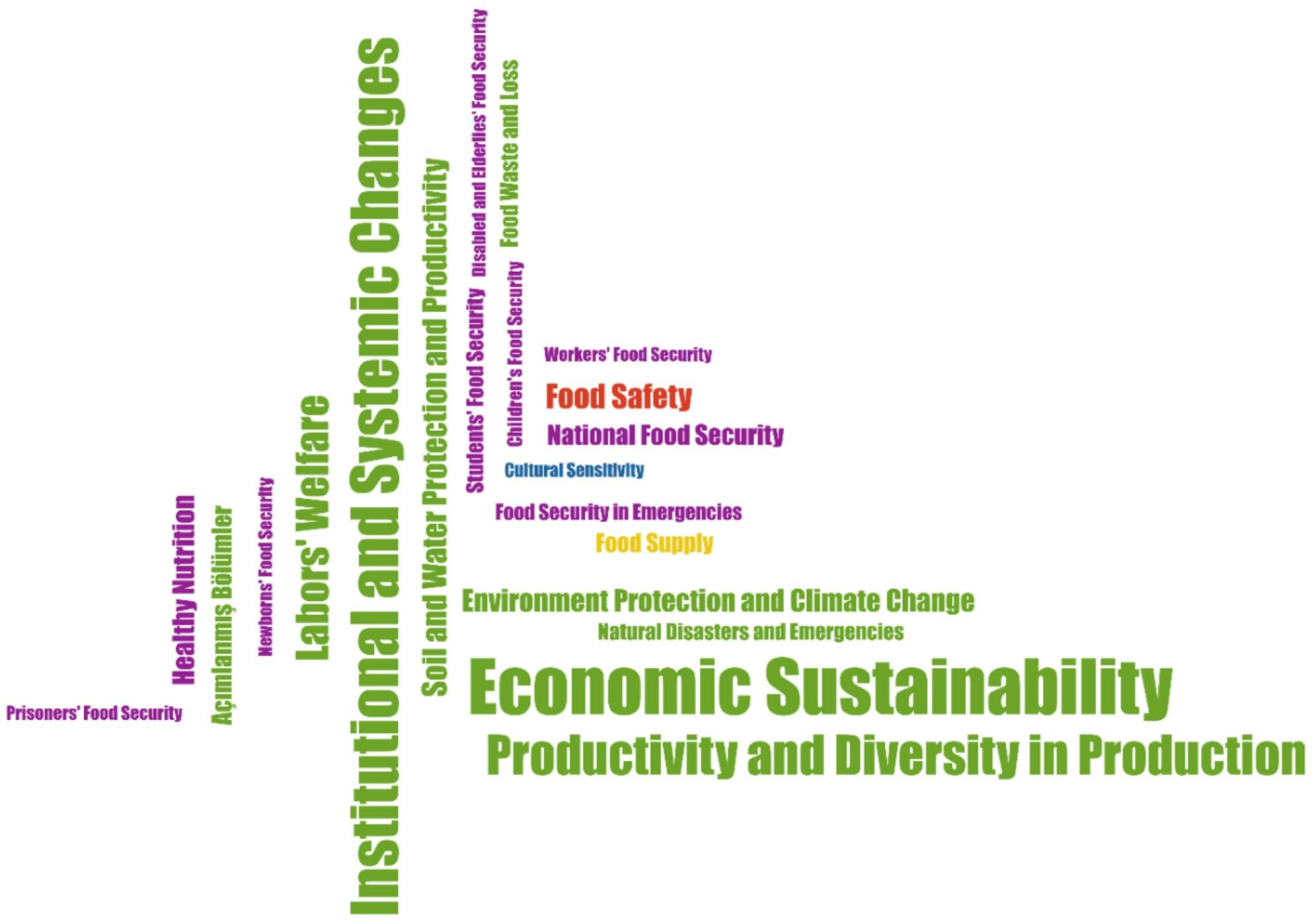
The code cloud of the PHN framework

The document analysis revealed that PHN themes were found in the documents of 32 out of 44 parties. This data obtained were categorized into five main themes which were further divided into six categories, which were finally subdivided into 15 specific codes (Supplementary Table 2). Our analysis ranked frequency of these codes, in order to determine which themes were most promoted in the documents. As depicted with the code cloud derived from MAXQDA in Fig. 1, after sustainable agriculture, the majority of policies were produced in the theme of food security, followed by food safety, food supply, and cultural sensitivity. Moreover, the category of sustainable nutrition was not used in the documents of any party or alliance.

**Fig. 2:**
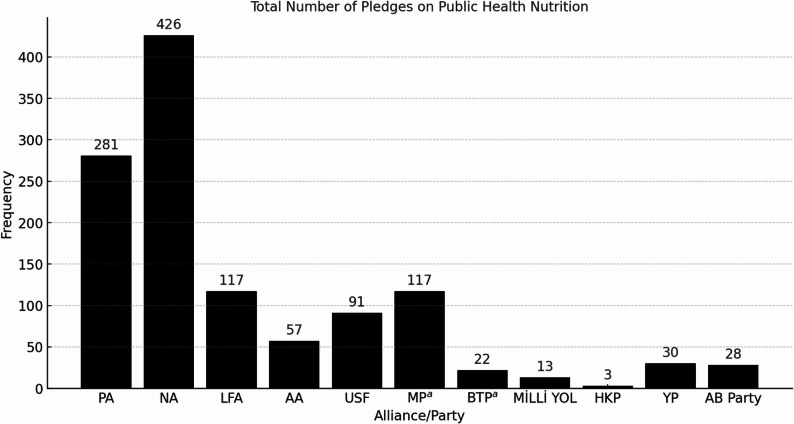
The illustration of the frequency of the PHN-related pledges found in documents

Overall, documents were coded 1209 times with the PHN framework. Each party/alliance’s number of codes are given in Supplementary Table 2. The alliances/parties, from having the highest number of pledges to the lowest, are as follows (Fig. 2): The NA, PA, LFA, MP^a^, USF, AA, YP, AB Party, VP, BTP^a^, MİLLİYOL, and HKP. The NA and PA have been the alliances that promised more comprehensive pledges compared to other alliances.

The themes/categories/codes regarded as the most important ones based on Türkiye’s specific issues mentioned above were healthy nutrition; workers’ and students’ food security; food security in emergencies; natural disasters and emergencies; cultural sensitivity; institutional and systemic changes; productivity and diversity in production; food waste and loss; food supply; and food safety. In the following section, these themes and codes are presented in detail. Each one is described in terms of its core content and defining characteristics. Additionally, the key proponents and perspectives associated with each theme are discussed to provide a comprehensive understanding of their significance.

### Nutrition security

The nutrition security theme encompasses the categories of food security, healthy nutrition, and sustainable nutrition with sub-codes. The analysis revealed that political parties and alliances approached nutrition security through varying emphases, which reflect differing understandings of the relationship between food, health, and social welfare.

Specifically, the NA and PA produced the highest number of policies within healthy nutrition category. While the NA and PA both promoted nutrition security, the PA does not pursue this advocacy through legislative initiatives for dietitians. The NA and AA focused on professional legislation for dietitians (Supplementary Table 5).

*“We will revise and implement the National Healthy Nutrition and Active Life Program to prevent childhood obesity and launch exclusive programs at preschool*,* primary*,* and secondary school levels.”* (The NA) [39].

While an interest in the broader theme of nutrition security was prevalent in the findings, different alliances and parties emphasized subthemes— the students’ food security, workers’ food security, and food security in emergencies—at varying quantitative levels.

The students’ food security code includes providing healthy foods in school cafeterias and student dormitories and ensuring nutritious meals. The LFA has been the alliance that paid the greatest attention to this area by emphasizing access-oriented measures. These measures, which linked food security directly to poverty alleviation and social equity, include providing free meals for students across all levels of education (Supplementary Table 5).

*“We will provide free meal services to students at all levels of education.”* (The LFA) [40].

The workers’ food security code indicates meeting the nutritional needs of workers. The NA, PA, and LFA have each promised one policy in this area (Supplementary Table 5).

*“We will develop solutions to address the issues faced by agricultural workers*,* such as wages*,* working hours*,* occupational safety*,* health*,* social security*,* transportation*,* housing*,* nutrition*,* access to clean water*,* sanitation*,* and children’s education.”* (The LFA) [40].

The food security in emergencies code encompasses measures like emergency plans, food stockpiling, and restructuring distribution systems. Policies have been developed primarily by the NA, followed by the LFA, PA, and AA, emphasizing structural vulnerabilities in Türkiye’s food supply during crises. Other parties or alliances produced no policies in this area, reflecting a narrow conceptualization of food security limited to peacetime or economic contexts rather than comprehensive resilience planning (Supplementary Table 5).

*“We will establish agricultural and food hubs near major metropolitan areas to minimize the risk of food access during disasters*,* crises*,* and pandemics.”* (The NA) [39].

### Sustainable agriculture

Sustainable agriculture emerged as a critical theme with several sub-categories indicating different dimensions of sustainability in agriculture. The NA and PA produced the highest number of codes in this field.

The natural disasters and emergencies code indicates the need to ensure the sustainability of agricultural production during and after natural disasters and emergencies. The NA, PA, and AB Party have been the ones that paid the greatest attention to this code, criticizing lack of sustainable measurements (Supplementary Table 5).

*“It is essential to procure and allocate tractors*,* plows*,* and similar agricultural machinery*,* tools*,* and other equipment that have been damaged or rendered unusable due to being buried under rubble. The costs should be partially covered by the government and partially recovered through long-term*,* favorable leasing arrangements.”* (The NA) [41].

Productivity and diversity in the production code involves using agricultural techniques that yield higher productivity, producing native seeds, planting different types of crops, and increasing production diversity. In this area, the PA has been the alliance promising the majority of pledges, emphasizing national competitiveness and self-sufficiency (Supplementary Table 5).

“We will establish Specialized Seed Production Zones to promote and develop local and national seed cultivation.” (The PA) [42].

The food waste and loss code includes certain measures such as preventing losses in the food supply chain. The MP^a^ has been the party promising most of pledges, adopting a community-oriented and state-led framework for planned agricultural production (Supplementary Table 5). This framing uniquely integrates sustainability with social responsibility, aligning more closely with SDG 12 on responsible consumption and production.

“*To prevent crops from being left unused in the hands of farmers or abandoned in the fields*,* and to ensure that time*,* effort*,* capital*,* and knowledge resources are not wasted*,* we will implement state-led*,* public*,* and community-focused policies in agriculture and pursue the transition to planned agricultural production.”* (The MP^a^) [43].

The environmental protection and climate change code includes promoting biodiversity, reducing the use of chemical fertilizers and pesticides, adopting soilless agriculture, producing biogas, using fermented fertilizers, exploring alternative production methods, and utilizing renewable energy sources. The NA, followed by the PA, produced most of pledges without deeper consideration of the social and public health implications (Supplementary Table 5).

*“We will establish biogas facilities in 41 provinces within the scope of the Livestock Agriculture Organized Zone.”* (The NA) [44].

The institutional and systemic changes code involves providing training to farmers, developing control mechanisms, digitalization and artificial intelligence applications, R&D studies, and institutional changes. The NA produced the biggest number of policies in this area, focusing on institutional restructuring (Supplementary Table 5).

*“We will restructure the Ministry of Agriculture and Forestry as the Ministry of Agriculture and Food.”* (The NA) [45].

### Food safety

The food safety theme includes the implementation of hygiene standards in food production, processing, and storage, as well as the control of harmful chemicals and pathogens. The NA produced the majority of pledges, followed by the PA (Supplementary Table 5).

*“We will reestablish the abolished Biosafety Board.”* (The NA) [39].

### Food supply

The food supply theme involves maintaining adequate levels of agricultural production, ensuring the efficient operation of logistics and distribution systems, and timely delivery of food products to markets. The PA has been the alliance promising the highest number of pledges, followed by the NA (Supplementary Table 5).

*“We will establish a Supply Security Monitoring System to track the adequacy of strategic products.”* (The PA) [42].

### Cultural sensitivity

The least developed theme was cultural sensitivity, addressed only by the NA and PA (Supplementary Table 5). The cultural sensitivity theme indicates promoting respect for cultural diversity and the recognition and continuity of food culture. The PA’s reference to halal food regulations demonstrates a narrow, religion-based understanding of cultural aspects of nutrition, rather than a broader recognition of Türkiye’s diverse food traditions and their potential role in promoting sustainable and healthy diets.

*“Considering that the majority of the population is Muslim*,* regulations should be made regarding halal food and halal slaughter.”* (The PA) [46].

B. Sustainable development goals

**Fig. 3:**
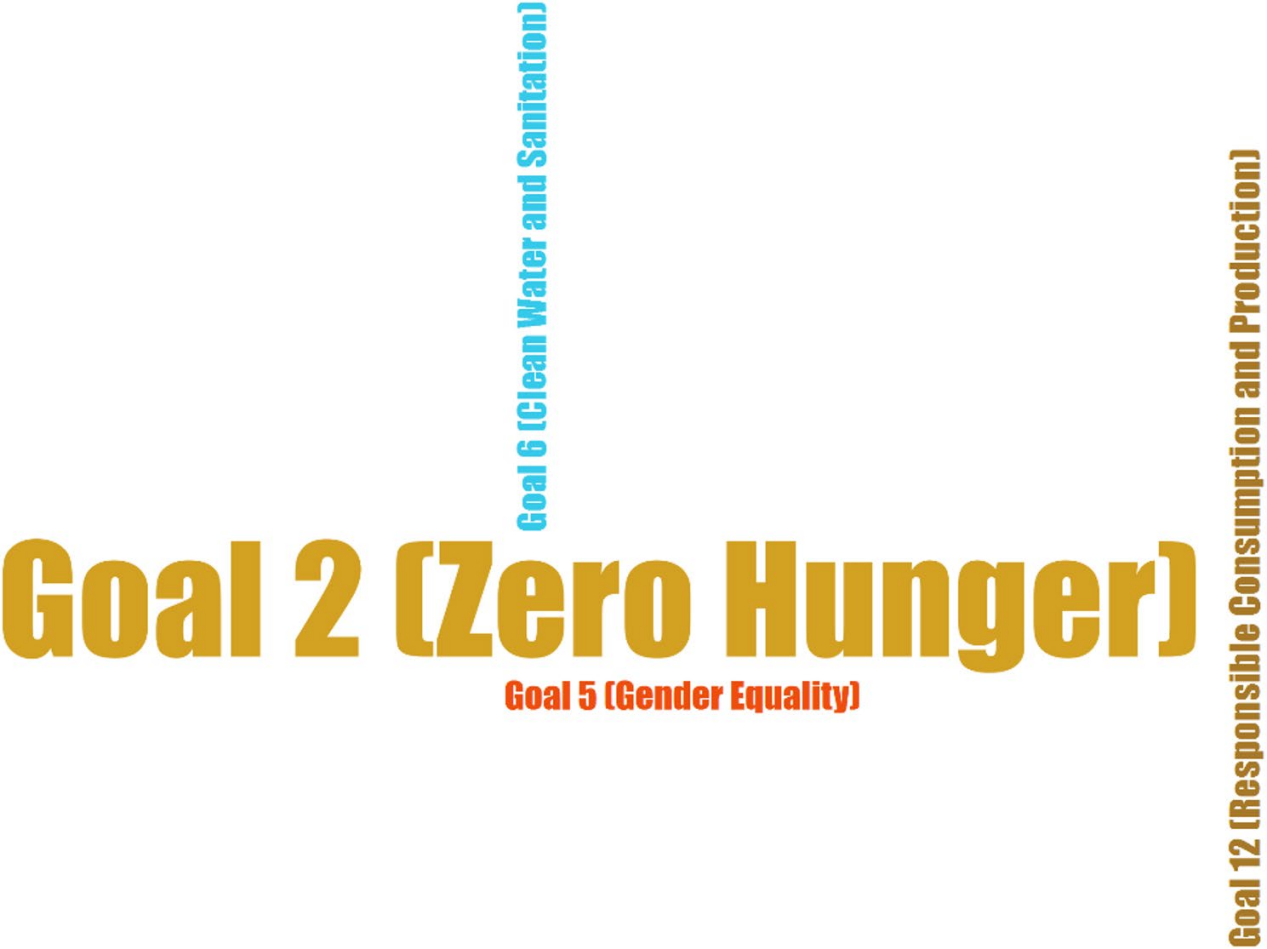
The code cloud of SDGs derived from MAXQDA

**Fig. 4:**
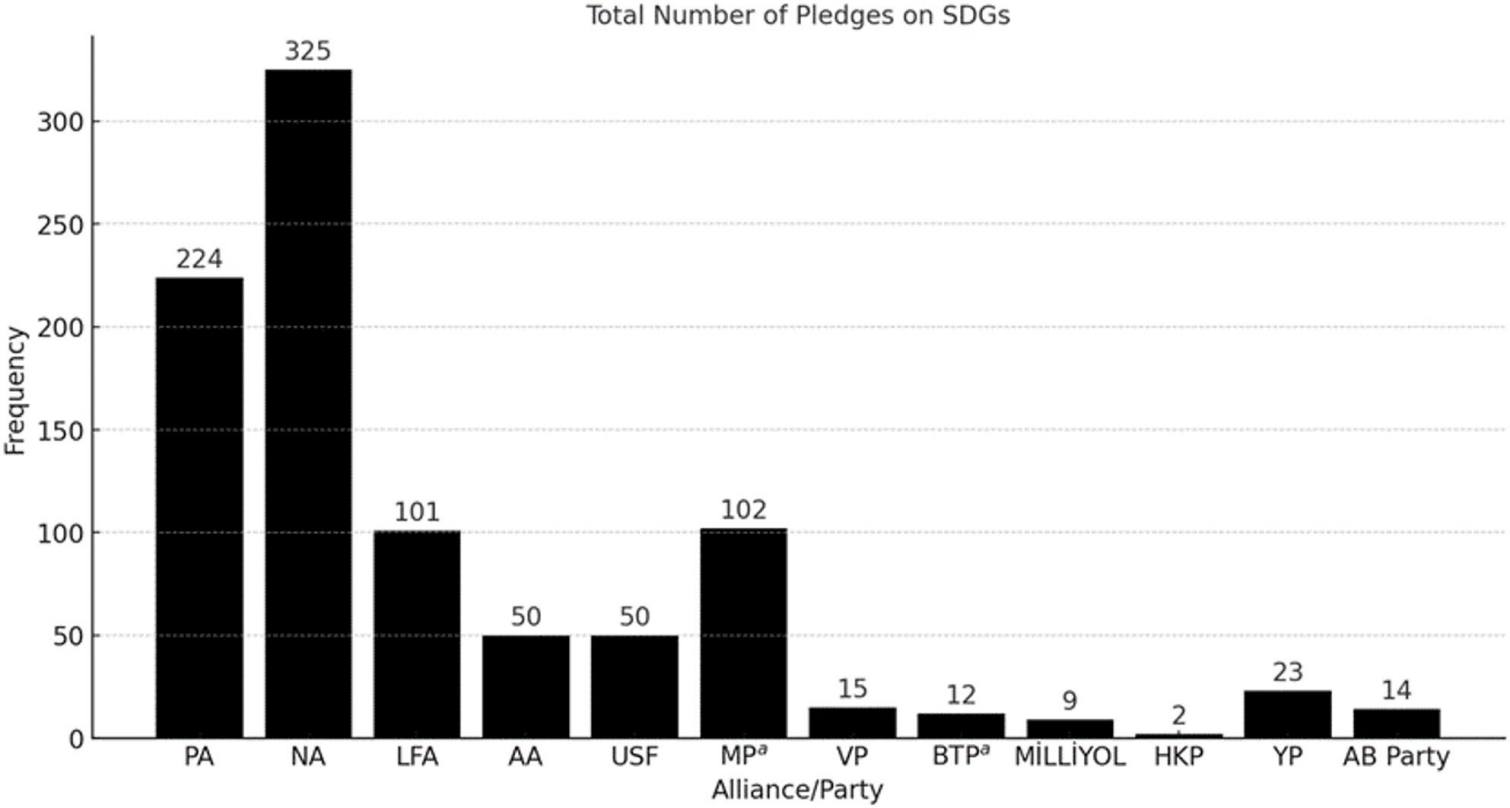
Illustration of the frequency of the SDG-related pledges

Within the SDGs framework, 941 codes were generated. The NA’s documents have the highest number of codes, totaling 325. The analysis of SDG-related content showed a quantitative predominance of mentions to Goal 2 (Zero Hunger). In this regard, Goal 2 (Zero Hunger) was the most coded theme, totaling 892 times. Goal 5 (Gender Equality), Goal 6 (Clean Water and Sanitation), and Goal 12 (Responsible Consumption and Production) themes were coded 14, 8, 27 times respectively (Fig. 3). The alliances/parties, from having the highest number of pledges to the lowest in this context are as follows (Fig. 4): The NA, PA, MP^a^, LFA, USF, AA, YP, VP, AB Party, BTP^a^, MİLLİYOL, HKP. Each party/alliance’s code numbers in SDGs are given Supplementary Figs. 1,2,3, and 4. Those which did not give any pledges are not included in the figures.

The overwhelming focus on Goal 2 suggests that most parties conceptualized nutrition and food systems primarily through the lens of hunger eradication and agricultural productivity, rather than as part of a broader sustainability agenda. The limited attention to Goals 5, 6, and 12 indicates gaps in integrating gender, water management, and sustainable consumption dimensions into nutrition policy frameworks.

### Goal 2: zero hunger

This goal refers to ending hunger, achieving food security and improved nutrition, and promoting sustainable agriculture. The NA has been the alliance promising the most pledges in this area (Supplementary Table 6).

*“We will ensure food safety and food security from farm to table.”* (The NA) [39].

### Goal 5: gender equality

This goal refers to gender equality in society and empowering all women and girls. Equality and the social dimension must be considered in food security policies. The LFA has been the alliance promising the majority of pledges (Supplementary Table 6). Its reference to gender equity for female agricultural workers reflects a social justice-oriented interpretation of nutrition and food systems. On the other hand, the NA adopts a development-oriented strategy to strengthen women’s roles in modern agricultural systems.

*“We will implement the principle of positive discrimination for female seasonal agricultural workers and ensure that local governments take responsibility.”* (The LFA) [40].

### Goal 6: clean water and sanitation

This goal refers to ensuring accessible water and wastewater services for all and sustainable water management. The NA has been the alliance promising the most pledges (Supplementary Table 6).

*“We will ensure to protect and improve drinking water sources*,* provide clean water to residential areas*,* and enable citizens to drink water directly from their taps at home.”* (The NA) [39].

### Goal 12: responsible consumption and production

This goal refers to promoting sustainable consumption and production habits in agriculture. The ones that paid the greatest attention to were the NA and MP^a^ (Supplementary Table 6). While the MPa emphasizes consumer-level behavioral change through awareness campaigns to reduce household food waste, the NA focuses on system-level interventions by identifying loss points along the food supply chain.

*“We will implement state-led*,* public-oriented*,* and community-focused policies in agriculture to prevent products from being wasted by farmers or in the fields*,* and to avoid wasting time*,* labor*,* capital*,* and knowledge resources. We will foster the transition to planned agricultural production.”* (The MP^a^) [43].

## Discussion

This study analyzed the extent and diversity of the emphasis on PHN as demonstrated by political parties and alliances for the 2023 Türkiye Election. It also analyzed the alignment of policies with the SDG’s overlapping targets.

Türkiye’s political landscape in the 2023 General Election was structured around several major alliances reflecting distinct ideological orientations. The PA, led by the ruling party, the AKP, primarily represents a conservative, nationalist, and right political block. The main opposition, the NA, led by the main opposition party, the CHP, brought together parties ranging from center-left to center-right, positioning itself broadly within a social democratic and liberal democratic framework. In addition, the LFA reflects a left-wing and pro-labor orientation, while smaller alliances such as the AA and the USF represent nationalist and socialist ideological positions, respectively [47, 48].

The qualitative analyses demonstrated that the political parties and alliances focused mostly on the theme of sustainable agriculture by varying extents. The NA and PA produced greater numbers and broader frameworks of policies compared to other alliances/parties. Given that the PA and NA included the governing party and the main opposition party - the actors receiving the highest shares of the vote- it is expected that they produced a higher number of pledges. As the primary competitors for government, they were more likely to present extensive and detailed policy commitments. The MP^a^ was identified as the party that proposed the majority of policies among non-aligned parties, while the AA and USF were generally inadequate in policy proposals. It is noteworthy that the category of sustainable nutrition has not been used in the documents of any party or alliance. Policies related to relevant targets under the SDGs were generally less emphasized. Some parties/alliances including the NA, LFA, and MP^a^ conducted a detailed analysis of Türkiye’s current situation in the context of nutrition, food, and agriculture while developing their pledges.

Türkiye has a significant lack of dietitian profession legislation and comprehensive policies addressing nutrition-related diseases. The fact that the dietitian profession legislation has been proposed by only two alliances, the NA and AA, is a drawback. Standardization, recognition, and the rights of dietitians are not properly regulated yet, resulting in performing this profession by so-called life coaches and other people who lack the required education [49, 50]. This lack of proper regulation not only undermines the credibility of dietetic profession but also poses risks to public health, as individuals may receive inadequate or harmful dietary advice. Moreover, although there are pledges related to obesity and diabetes, they have been excluded from this study since most of these do not address PHN. Obesity and other nutrition-related diseases could also be managed or prevented through non-nutrition-related approaches at the macro level [51]. Therefore, apart from the themes included in this study, it is critical that social, economic, and environmental policies are also developed to be integrated to improve PHN and lift people out of the vicious poverty cycle by providing sustainable solutions [52].

Evaluating pledges in terms of SDGs, which enabled us to locate policy pledges in a global context, demonstrated their insufficiency. While Goal 2 (Zero hunger) received some attention, it was still inadequate in terms of eradicating the root causes, particularly poverty. Moreover, Goals 5, 6, and 12 were almost entirely neglected, highlighting gaps in addressing PHN within the framework of the SDGs. Goal 2 is crucial for Türkiye as 16.2% of Turkish people reported experiencing food insecurity and 23.4% of them fear of not having enough food, according to the TNHS [53]. Moreover, Turkish women have been facing gender inequality in terms of salary, ownership, and insurance in working in agriculture [54, 55]. Studies show that accessing clean drinking water, is emphasized in Goal 6, is an issue threatening health by containing toxic and carcinogenic chemicals in important drinking and irrigation water resources in Türkiye [56, 57]. When it comes to the responsible consumption and production targeted by Goal 12, according to the 2018 Waste Report of Türkiye, 26 million tonnes of food were wasted annually in Türkiye [58].

According to the election results, the PA ranked first with 49.47% of the total votes, and the NA ranked second with 35.02% of the total votes. The LFA received 10.55% of the total votes, while the AA received 2.43% and the USF received 0.29% [59].

The parties forming the alliances were seen to have diverse political orientations creating conflicts for them. Although the center left-wing Democratic Left Party (DSP, Demokratik Sol Parti) has been in opposition for many years and has had an anti-AKP stance, it was part of the PA in the 2023 General Election. Therefore, in the current study, the DSP’s documents were analyzed under the PA. Similarly, although the Felicity Party (SP, Saadet Partisi) is a right-wing party, it joined the NA. In this regard, it is unknown that to what extent the parties in the alliance do agree on policies without a joint policy statement. For alliances with a joint policy statement, it was assumed that there was an agreement on the policies. Moreover, contributions from the members in the alliances regarding proposed policies were not distributed equally. The coded sections mostly belong to certain parties’ documents. Although the alliance members’ efforts remained uneven, the alliances were still assessed as a whole. Furthermore, the fact that some parties’ programs (e.g. DSP) were published almost ten years ago with no further modification makes the results questionable. Indeed, it was seen that some party programs pledged to establish existing institutions and used the former names of the state institutions.

The pledges demonstrate class antagonisms that have characterized the political parties. In this sense, even right-wing parties were compelled to offer left-leaning promises, given that the majority of voters belong to the working class. The frequent use of religious motifs, such as Islamic symbols, and references to specific identities, including Kurdish, has been noted. This raises concerns about the possibility of discriminatory practices. The main shortcomings of these pledges are their vagueness and lack of detail and clarity. The use of vague language such as “will be revised/evaluated/improved/encouraged” and not indicating how these revisions, evaluations or improvements will be conducted is a clear example. Additionally, some prohibition pledges fail to provide any alternatives for the things they prohibit, contributing to the ambiguity of these promises. This could be a reason why politicians are often able to claim later that they have fulfilled most of their promises.

Furthermore, some documents (e.g., the AKP, CHP, DEVA, İYİP, YSP) used active language like “we will do…”, while others (e.g., the ZP, SP, MHP) preferred passive language like “…should/must be done”. This may influence readers’ perceptions of the agency and accountability of politicians [60]. Additionally, the readability of certain documents was challenging. Some parties have created documents with small font sizes, complex formats, and grammatical and spelling mistakes [61]. This can reduce readability and potentially hinder voter engagement, and therefore, there is room for improvement regarding reaching wider audiences.

Voter decision-making involves various determinants, and one key factor is the extent to which parties’ pledges reach the electorate. This reach is influenced by disparities in media visibility, as some parties receive significantly more coverage than others. For example, many television news channels provide limited coverage of certain parties, which affects how effectively their pledges reach the voters, and although media bias itself is outside the scope of this study, this issue may help draw implications regarding the extent to which these pledges are communicated to the electorate [62].

Therefore, based on the election results, it is not possible to definitively conclude how many voters took PHN policies into account. However, the CHP, the main opposition party that won the local elections on 31 March 2024, established several low-cost municipal restaurants in Istanbul where a 4-course meal including a beverage was sold for around 1 USD in 2024. This could be considered as ecological evidence that policies in this area might play a role in the decision-making processes of voters [63, 64].

It is noteworthy that policies concerning refugees have been hardly put forward. In fact, only the NA and LFA developed such pledges. The increased anti-immigration sentiment in the midst of the global refugee crisis might encourage one to speculate that parties are formulating policies on these issues but not sharing them with the public, making the transparency of politicians questionable. For example, the AKP, which is the ruling party in the government, has already been conducting ongoing policies regarding refugees’ nutrition without any pledge in its document. Nevertheless, the NA and LFA have promised policies for workers and refugees [65].

Besides, the Turkish public has witnessed arguably interesting election strategies from political parties over the years. Some of them include the AKP throwing tea packages to the public at rallies, and the AKP and the CHP distributing boxes containing basic foodstuffs such as pasta, sugar, jam, salt, flour, and so on [66, 67]. When compared to pledges, these seemingly charities made with populist motives raise questions regarding whether politicians will implement comprehensive policy formulations to ensure sustainable PHN. Unlike charity-based food aid distributions, which could only offer temporary relief, various studies suggest that structural reforms, such as framing food security as a social right and improving workers’ wages, have a more lasting impact [68]. To illustrate, certain programs like the Supplemental Nutrition Assistance Program (SNAP) have successfully lifted many out of food insecure positions in the United States [69]. This underscores the need for policymakers to move beyond populist and short-term solutions, and prioritize sustainable policies that address the root causes of food insecurity.

Moreover, rather than focusing on the number of pledges and populist pledges, developing realistic and detailed policies may lead to more meaningful and effective outcomes. Given Türkiye’s recent crises, namely the 2018–2019 debt and currency crisis followed by the 2020 COVID pandemic, the capacity of alliances and parties to fulfill their policy promises is open to debate. Indeed, the conditions faced by the earthquake victims regarding lack of food security and food safety during the 2023 Türkiye-Syria earthquakes might have diminished trust in politicians’ ability to fulfill their promises. The results of the election demonstrated that the ruling AKP government lost votes in the earthquake zone but did not lose its place as the first party owing to the pledge for reconstruction of houses [70, 71]. Considering this contradictory electoral behavior, greater accountability regarding the feasibility and transparency of election promises should be further researched even though such accountability can only be applied to the political parties in government or ruling municipalities that implement their electoral pledges. The Twelfth Development Plan (2024–2028), which outlines Türkiye’s strategic priorities for social and economic development, published in 2023 by the Presidency, demonstrated that PHN-related policies could not find enough space. This confirms that sufficient importance was not given to this topic [72]. Therefore, it is essential to investigate the limited number of PHN strategies in the Twelfth Development Plan and assess to which extent these few initiatives are effectively implemented.

### Strengths and limitations

One of the most important strengths of this work is the analysis of written documents that are published on the parties’ websites, instead of social media or their public discourses. This ensures the reliability of its method by allowing a comprehensive examination of the parties’ promises. The other strength is that it contributed to literature as one of the pioneering research studies to be carried out on the subject. Political parties and policymakers can benefit from this study’s insights, which highlight the need for thorough analyses of policy proposals, including their feasibility, sustainability, and effectiveness. Consequently, this research is expected to serve as a valuable reference in both academic and policy-making contexts.

The limitation of this study is the bias arising from the subjective nature of qualitative research. The same data may be interpreted distinctly by different researchers and may lead to different results. Even though the research was conducted by both researchers and Turkish voters with arguably reducing professional distance, the data were analyzed the by three independent researchers to mitigate such potential bias.

The SDGs were used as a framework while analyzing pledges, as they provide a well-structured and generally accepted global model. However, no framework is perfect for addressing issues without any interest. Some researchers argue that the SDGs prioritize economic growth over environmental protection, and ironically, these practices may have negative impacts on vulnerable groups [73, 74].

## Conclusions

In conclusion, most of the parties/alliances remained inadequate in giving pledges in relation to PHN. This research provides significant insights into the prioritization of PHN and highlights the need for continued focus in this area to mitigate the rising economic burden of nutrition-related diseases and promote a healthier and more resilient population. It is necessary to develop clear, comprehensive, sustainable, and accountable policies that can address nutrition issues based on Türkiye, recognizing that public health nutrition is a critical subject. Future research should look at the degree of fulfilling their pledges and focus on longitudinal studies to evaluate the effectiveness of implemented nutrition policies and their impact on public health outcomes. Comparative studies between Türkiye and other countries can also provide valuable insights into best practices and innovative strategies for improving PHN.

### Research snapshot

#### Research question

To what extent do political parties in Türkiye reflect and prioritize public health nutrition and related SDGs in their election declarations, party programs, and formal website-based documents, demonstrating a broad understanding of nutrition-related public health issues?

#### Key findings

The political parties and alliances generally lacked a comprehensive understanding of nutrition-related public health issues such as obesity and diabetes for the context of Türkiye. Although the number of pledges was relatively higher in some themes, particularly concerning food insecurity, neither the alliances nor the political parties proposed sustainable and realistic pledges that target the root causes. Moreover, some pledges were ambitious such as eradicating hunger that these cannot even be realized after the next election period. Policies related to relevant targets under the Sustainable Development Goals (SDGs) were not emphasized adequately given the global importance of SDGs.

## Supplementary Information

Supplementary Material 1

Supplementary Material 2

Supplementary Material 3

Supplementary Material 4

Supplementary Material 5

## Data Availability

The documents analyzed during the current study are available from the corresponding author on reasonable request.

## Declarations

### **Declaration of AI and AI-assisted technologies in the writing process**

During the preparation of this work the authors used ChatGPT 4.o in order to get assistance in language editing and data visualization. After using this tool/service, the authors reviewed and edited the content as needed and take full responsibility for the content of the publication. 
