# Supplementary material for: A content analysis of 2023 Türkiye general election pledges on public health nutrition and related sustainable development goals
Source: J Health Popul Nutr. 2026 Apr 26;45:156. doi: 10.1186/s41043-026-01299-6 (PMC13289391; doi:10.1186/s41043-026-01299-6)
Supplement: Supplementary file 1 — Supplementary Material 1 [file 41043_2026_1299_MOESM1_ESM.docx]

**Supplementary Figure 1:** The illustration of frequency of pledges related to Goal 2: Zero Hunger found in parties/alliances’ document
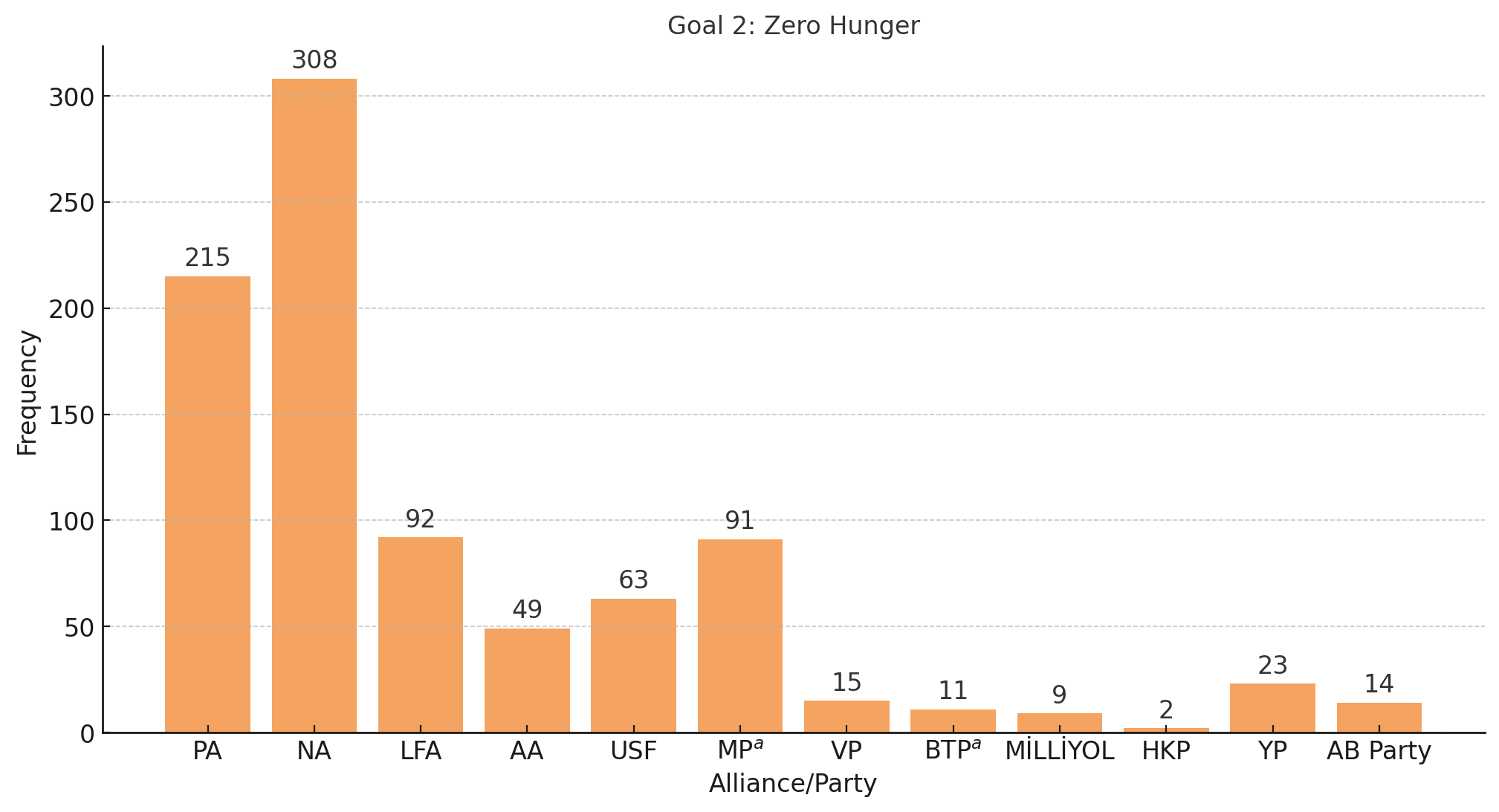
s.
