# Supplementary material for: A content analysis of 2023 Türkiye general election pledges on public health nutrition and related sustainable development goals
Source: J Health Popul Nutr. 2026 Apr 26;45:156. doi: 10.1186/s41043-026-01299-6 (PMC13289391; doi:10.1186/s41043-026-01299-6)
Supplement: Supplementary file 5 — Supplementary Material 5 [file 41043_2026_1299_MOESM5_ESM.docx]

**Supplementary Table 1**: Alliance Members and Non-Aligned Parties Participated in the 2023 General Election.

| **ALLIANCES** | **PARTIES** |
| --- | --- |
| People’s Alliance | Adalet ve Kalkınma Partisi (Justice and Development Party, AKP)  Milliyetçi Hareket Partisi (Nationalist Movement Party, MHP)  Yeniden Refah Partisi (New Welfare Party, YRP)  Büyük Birlik Partisi (Great Unity Party, BBP)  Demokratik Sol Parti (Democratic Left Party, DSP)  Hür Dava Partisi (Free Cause Party, HÜDAPAR) |
| Nation Alliance | Cumhuriyet Halk Partisi (Republican People's Party, CHP)  İyi Parti (Good Party, İYİP)  Saadet Partisi (Felicity Party, SP)  Demokrasi ve Atılım Partisi (Democracy and Progress Party, DEVA)  Gelecek Partisi (Future Party, GP)  Demokrat Parti (Democrat Party, DP) |
| Labour and Freedom Alliance | Halkların Demokratik Partisi (Peoples' Democratic Party, HDP)  Yeşiller ve Sol Gelecek Partisi (Green Left Party, YSP)  Türkiye İşçi Partisi (Workers’ Party of Türkiye)  Emek Partisi (Labour Party, EMEP)  Toplumsal Özgürlük Partisi (Social Freedom Party, TÖP)  Emekçi Hareket Partisi (Labourist Movement Party, EHP) |
| Ancestral Alliance | Zafer Partisi (Victory Party, ZP)  Adalet Partisi (Justice Party, AP)  Ülkem Partisi (My Country Party, ÜP)  Türkiye İttifak Partisi (Türkiye Alliance Party, TÜİP)  Güven Adalet ve Aydınlık Partisi (Trust, Justice and Enlightment Party, GAAP)  Vatan ve Hürriyet Partisi (Patriotic and Liberty Party, HÜRVATAN) |
| Union of Socialist Forces | Sol Parti (Left Party, SOL)  Türkiye Komünist Partisi (Communist Party of Türkiye)  Türkiye Komünist Hareketi (Communist Movement of Türkiye)  Devrim Hareketi (Revolutionary Movement, DH)  Türkiye Sosyalist İşçi Partisi (Socialist Workers' Party of Türkiye, TSİP)  Devrimci İşçi Partisi (Revolutionist Workers' Party, DİP)  Sosyalist Cumhuriyet Partisi (Socialist Republican Party, SCP) |
| **NON-ALIGNED PARTIES** | |
| Memleket Partisi (Homeland Party, MP^a^)  Anavatan Partisi (Motherland Party, ANAP)  Genç Parti (Young Party, GENÇPARTİ)  Bağımsız Türkiye Partisi (Independent Türkiye Party, BTP^a^)  Büyük Türkiye Partisi (Great Türkiye Party BTP^b^)  Adalet Birlik Partisi (Justice and Unity Party,AB Party)  Vatan Partisi (Patriotic Party, VP)  Güç Birliği Partisi (Power Union Party, GBP)  Yenilik Partisi (Innovation Party, YP)  Milli Yol Partisi (National Path Party, MİLLİ YOL)  Halkın Kurtuluş Partisi (People’s Liberation Party, HKP)  Millet Partisi (Nation Party, MP^b^)  Hak ve Özgürlükler Partisi (Rights and Freedom Party, HAK-PAR) | |

**Supplementary Table 2**: Pledge frequency according to the parties/alliances in Public Health Nutrition.*

| CODE System | | | Frequency | Alliance/Party | | | | | | | | | | | | |
| --- | --- | --- | --- | --- | --- | --- | --- | --- | --- | --- | --- | --- | --- | --- | --- | --- |
|  |  |  |  | PA | NA | LFA | AA | USF | MP^a^ | VP | BTP^a^ | MİLLİYOL | HKP | YP | AB-PARTY |  |
|  | | | 1209 | 281 | 426 | 117 | 57 | 91 | 117 | 24 | 22 | 13 | 3 | 30 | 28 |  |
| Themes | **Categories** | **Codes** |  |  |  |  |  |  |  |  |  |  |  |  |  |  |
| Nutrition Security |  |  |  |  |  |  |  |  |  |  |  |  |  |  |  |  |
|  | **Food Security** |  |  |  |  |  |  |  |  |  |  |  |  |  |  |  |
|  |  | **National Food Security** | 43 | 11 | 20 | 4 | - | 6 | 1 | 1 | - | - | - | - | - |  |
|  |  | **Prisoners' Food Security** | 1 | - | - | 1 | - | - | - | - | - | - | - | - | - |  |
|  |  | **Students' Food Security** | 18 | 1 | 5 | 9 | 1 | 1 | - | 1 | - | - | - | - | - |  |
|  |  | **Children's Food Security** | 10 | - | 5 | 3 | - | 1 | 1 | - | - | - | - | - | - |  |
|  |  | **Newborns' Food Security** | 3 | - | 3 | - | - | - | - | - | - | - | - | - | - |  |
|  |  | **Workers' Food Security** | 3 | 1 | 1 | 1 | - | - | - | - | - | - | - | - | - |  |
|  |  | **Disabled and Elderlies' Food Security** | 2 | - | 2 | - | - | - | - | - | - | - | - | - | - |  |
|  |  | **Food Security in Emergencies** | 12 | 2 | 6 | 3 | 1 | - | - | - | - | - | - | - | - |  |
|  | **Sustainable Nutrition** | | 0 | - | - | - | - | - | - | - | - | - | - | - | - |  |
|  | **Healthy Nutrition** | | 55 | 10 | 30 | 4 | 2 | 3 | 3 | - | 3 | - | - | - | - |  |
| Food Safety | | | 66 | 15 | 35 | 4 | 3 | 1 | 4 | - | 1 | 1 | - | 1 | 1 |  |
| Food Supply | | | 30 | 14 | 6 | 1 | 1 | 2 | 4 | - | 1 | - | - | 1 | - |  |
| Sustainable Agriculture | | |  |  |  |  |  |  |  |  |  |  |  |  |  |  |
|  | **Changes in Work and Labor Factors** | |  |  |  |  |  |  |  |  |  |  |  |  |  |  |
|  |  | **Economic Sustainability** | 274 | 63 | 92 | 23 | 14 | 21 | 33 | 3 | 6 | 2 | - | 7 | 10 |  |
|  |  | **Labors' Welfare** | 122 | 19 | 22 | 31 | 3 | 16 | 15 | 6 | 3 | - | - | 6 | 1 |  |
|  | **Changes in Agricultural Practices** | |  |  |  |  |  |  |  |  |  |  |  |  |  |  |
|  |  | **Productivity and Diversity in Production** | 169 | 54 | 45 | 4 | 12 | 9 | 24 | 3 | 4 | 5 | - | 4 | 5 |  |
|  |  | **Food Waste and Loss** | 24 | 5 | 6 | 2 | 1 | - | 9 | - | 1 | - | - | - | - |  |
|  |  | **Soil and Water Protection and Productivity** | 67 | 20 | 23 | 7 | 4 | 8 | 1 | - | 1 | - | 1 | 2 | - |  |
|  |  | **Environment Protection and Climate Change** | 55 | 15 | 22 | 9 | - | 4 | 4 | - | - | - | - | 1 | - |  |
|  |  | **Natural Disasters and Emergencies** | 37 | 11 | 16 | - | 2 | 2 | - | 1 | - | - | - | - | 5 |  |
|  | **Institutional and Systemic Changes** | | 213 | 39 | 84 | 11 | 13 | 16 | 18 | 9 | 2 | 5 | 2 | 8 | 6 |  |
| Cultural Sensitivity |  | | 3 | 1 | 2 | - | - | - | - | - | - | - | - | - | - |  |

*: Only parties whose pledges have been reached through publicly available documents are included in the table.

PA: People’s Alliance (Cumhur İttifakı)

NA: Nation Alliance (Millet İttifakı)

LFA: Labour and Freedom Alliance (Emek ve Özgürlük İttifakı)

AA: Ancestral Alliance (Ata İttifakı)

USF: Union of Socialist Forces (Sosyalist Güç Birliği)

MP^a^: Memleket Partisi (Homeland Party)

VP: Vatan Partisi (Patriotic Party)

BTP^a^: Bağımsız Türkiye Partisi (Independent Türkiye Party)

MİLLİYOL: Milli Yol Partisi (National Path Party)

HKP: Halkın Kurtuluş Partisi (People’s Liberation Party)

YP: Yenilik Partisi (Innovation Party)

AB Party: Adalet Birlik Partisi (Justice and Unity Party)

**Supplementary Table 3**: Specific Targets of Sustainable Development Goals related to Public Health Nutrition.^32^

| Sustainable Development Goals | Related Targets |
| --- | --- |
| Goal 2 | -By 2030, end hunger and ensure access by all people, in particular the poor and people in vulnerable situations, including infants, to safe, nutritious and sufficient food all year round  -By 2030, end all forms of malnutrition, including achieving, by 2025, the internationally agreed targets on stunting and wasting in children under 5 years of age, and address the nutritional needs of adolescent girls, pregnant and lactating women and older persons  - By 2030, double the agricultural productivity and incomes of small-scale food producers, in particular women, indigenous peoples, family farmers, pastoralists and fishers, including through secure and equal access to land, other productive resources and inputs, knowledge, financial services, markets and opportunities for value addition and non-farm employment  - By 2030, ensure sustainable food production systems and implement resilient agricultural practices that increase productivity and production, that help maintain ecosystems, that strengthen capacity for adaptation to climate change, extreme weather, drought, flooding and other disasters and that progressively improve land and soil quality  - By 2020, maintain the genetic diversity of seeds, cultivated plants and farmed and domesticated animals and their related wild species, including through soundly managed and diversified seed and plant banks at the national, regional and international levels, and promote access to and fair and equitable sharing of benefits arising from the utilization of genetic resources and associated traditional knowledge, as internationally agreed  - Increase investment, including through enhanced international cooperation, in rural infrastructure, agricultural research and extension services, technology development and plant and livestock gene banks in order to enhance agricultural productive capacity in developing countries, in particular least developed countries  - Correct and prevent trade restrictions and distortions in world agricultural markets, including through the parallel elimination of all forms of agricultural export subsidies and all export measures with equivalent effect, in accordance with the mandate of the Doha Development Round  -Adopt measures to ensure the proper functioning of food commodity markets and their derivatives and facilitate timely access to market information, including on food reserves, in order to help limit extreme food price volatility |
| Goal 5 | - Undertake reforms to give women equal rights to economic resources, as well as access to ownership and control over land and other forms of property, financial services, inheritance and natural resources, in accordance with national laws. (Indicators of this target are: (a) Proportion of total agricultural population with ownership or secure rights over agricultural land, by sex; and (b) share of women among owners or rights-bearers of agricultural land, by type of tenure |
| Goal 6 | - By 2030, improve water quality by reducing pollution, eliminating dumping and minimizing release of hazardous chemicals and materials, halving the proportion of untreated wastewater and substantially increasing recycling and safe reuse globally  - By 2030, achieve universal and equitable access to safe and affordable drinking water for all |
| Goal 12 | - By 2030, halve per capita global food waste at the retail and consumer levels and reduce food losses along production and supply chains, including post-harvest losses  - By 2020, achieve the environmentally sound management of chemicals and all wastes throughout their life cycle, in accordance with agreed international frameworks, and significantly reduce their release to air, water and soil in order to minimize their adverse impacts on human health and the environment |

**Supplementary Table 4:** Pledge frequency according to the parties/alliances in Sustainable Development Goals.*

| SDG’s/ Parties | Total | PA | NA | LFA | AA | USF | MP^a^ | VP | BTP^a^ | MİLLİYOL | HKP | YP | ab pARTY |
| --- | --- | --- | --- | --- | --- | --- | --- | --- | --- | --- | --- | --- | --- |
| G2^a^ | 892 | 215 | 308 | 92 | 49 | 63 | 91 | 15 | 11 | 9 | 2 | 23 | 14 |
| G5^b^ | 14 | 2 | 3 | 6 | - | 1 | 2 | - | - | - | - | - | - |
| G6^c^ | 8 | 2 | 5 | 1 | - | - | - | - | - | - | - | - | - |
| G12^d^ | 27 | 5 | 9 | 2 | 1 | - | 9 | - | 1 | - | - | - | - |
| Total |  | 224 | 325 | 101 | 50 | 64 | 102 | 15 | 12 | 9 | 2 | 23 | 14 |

*: Only parties whose pledges have been reached are included in the table.

^a^Goal 2: Zero Hunger, ^b^Goal 5: Equality, ^c^Goal 6: Clean Water Sanitation, ^d^Goal 12: Responsible Consumption and Production

PA: People’s Alliance (Cumhur İttifakı)

NA: Nation Alliance (Millet İttifakı)

LFA: Labour and Freedom Alliance (Emek ve Özgürlük İttifakı)

AA: Ancestral Alliance (Ata İttifakı)

USF: Union of Socialist Forces (Sosyalist Güç Birliği)

MP^a^: Memleket Partisi (Homeland Party)

VP: Vatan Partisi (Patriotic Party)

BTP^a^: Bağımsız Türkiye Partisi (Independent Türkiye Party)

MİLLİYOL: Milli Yol Partisi (National Path Party)

HKP: Halkın Kurtuluş Partisi (People’s Liberation Party)

YP: Yenilik Partisi (Innovation Party)

AB Party: Adalet Birlik Partisi (Justice and Unity Party)

**Supplementary Table 5**: Pledges given by parties/alliances in Public Health Nutrition.*

| Themes | | Codes | Quotes |
| --- | --- | --- | --- |
| Nutrition Security | | Healthy Nutrition | “*We will define the job descriptions, authorities, and responsibilities of physicians, dentists, pharmacists, psychologists, dietitians, physiotherapists, nurses, and new healthcare professions*.” (The NA)^71^  “*We will enhance our practices and inspections to make the information on food packaging more visible and easier to understand within the scope of healthy nutrition. By 2028, we will double the proportion of Nutrition-Friendly Schools among White Flag Schools.*” (The PA)^72^  “*We will establish accessible food banks to ensure children's right to adequate, balanced, and healthy nutrition*.” (The LFA)^73^  “*Agriculture and livestock sectors should be restructured based on public ownership, external dependency should be ended, and Türkiye must be able to provide all its citizens with affordable, quality, and healthy food.*” (The USF)^74^    “*With the policies we will implement, we will create an agricultural sector that is based on adequate and balanced nutrition for the community, utilizes advanced technology, information, and data, addresses infrastructure issues, has high organization and efficiency, engages in planned production with supply and demand balance, enhances international competitiveness, and uses natural resources (soil, water basins, water sources) in a sustainable manner*.” (The MP^a^)^42^  “*Especially for auxiliary health professional groups like psychologists and dietitians who face issues with their Professional Law, the preparation of new professional laws and the updating and regulation of existing laws will be carried out under the leadership and guidance of this 'Consultative Board,' ensuring that these professional groups will have their working areas organized as requested.*” (The AA)^75^  “*To raise awareness about healthy living, we will facilitate easy access to sports facilities through public transportation, reduce transportation barriers, and create awareness through healthy nutrition campaigns*.” (The BTP^a^)^76^ |
|  | | Students’ food security | “*We will build new state dormitories as advanced social living spaces, where students are seen as adult citizens rather than children requiring supervision. These dormitories will provide psychosocial support, host social and cultural activities, and offer healthy, high-quality, and free nutrition services.*” (The LFA)^77^  “*We will provide healthy, nutritious, and delicious meals free of charge in the dormitories.*” (The NA)^71^  “*We will provide free education at every stage. Not only will school, books, and stationery be free, but also meals, transportation, and accommodation will be provided at no cost.*” (The PA)^78^  “*All needs of successful young people who continue or struggle to continue their education under difficult economic conditions, including accommodation, food, and supplies, will be met.*” (The AA)^79^  “*The state will cover the nutrition, clothing, civilized recreation, and cultural needs of students who are studying away from their families, as well as those who are orphaned or in need of assistance.*” (The VP)^80^  “*The state covers the accommodation and nutrition needs of students who are studying away from their families, as well as those who are orphaned or in need of assistance.*” (The USF)^81^ |
|  | | Workers’ food security | “*We will develop solutions to address the issues faced by agricultural workers related to wages, working hours, occupational safety, health, social security, transportation, housing, nutrition, access to clean water, sanitation, and children's education.*” (The LFA)^73^  “*By collaborating with local governments, we will implement the 'Seasonal Living Spaces' project within the next 2 years to improve the living standards of seasonal agricultural workers, focusing on accommodation, health, and cleanliness. We will also cover the transportation and nutrition costs for their children to enable them to attend the nearest educational institution.*” (The NA)^71^  “*The state should not tax minimum wage earners, and basic necessities should also be exempt from taxes. The current definition of the minimum wage in Regulation No. 25540, which states that the minimum wage is 'the amount paid to workers for a normal working day that is sufficient to cover the worker's essential needs such as food, housing, clothing, health, transportation, and culture at the minimum level according to current prices,' should be revised to include the worker's family in this definition.*” (The PA)^45^ |
|  | | Food Security in Emergencies | “*After a disaster, a mobile pharmacy network should be planned to work in coordination with relevant NGOs and donors of medicines, vaccines, and food supplements.*” (The NA)^40^  “*First and foremost, accurate damage assessment should be conducted in the cities affected by the earthquake, and the shelter and nutrition needs of the earthquake victims must be regularly met. It is essential to create healthy and humane living spaces and urgently allocate all available resources, including the country's vacant housing stock and public social facilities, to meet the needs of the earthquake-affected population.*” (The LFA)^82^  “*We will support projects related to research and development and hygienic storage technologies for providing shelter, food, and water to disaster survivors.*” (The PA)^72^  “*To reduce the social and psychological damage of all types of disasters, the requirements of the social welfare state will be fulfilled without discrimination among citizens, and all barriers to accessing basic health services, food security and nutrition, education, and housing will be removed.*” (The AA)^79^ |
| Sustainable Agriculture | | Natural disasters and Emergencies | “*Significant damage has occurred in deep wells, particularly in Hatay, Gaziantep, and Kilis. To address the irrigation needs, equipment brought from outside the region should be used for drilling support or repairs. Additionally, considering the drought issues specific to 2023, support should be provided for drilling, technical assistance, and materials, including well casing, submersible pumps, and electrical installations, to ensure the continuity of irrigation.*” (The NA)^40^  “*15% of our country's agricultural output and one-seventh of our farmers are located in the provinces affected by the recent earthquake disasters. We will implement a robust recovery program to maximize the potential of these provinces after the disaster.*” (The PA)^72^  “*For all plant products, the requirement to be registered in the Farmer Registration System will be established for economically productive fruit trees and vines, including their seedlings. We will ensure that damages caused by risks such as hail, storms, tornadoes, fires, earthquakes, landslides, floods, vehicle collisions, and snow load are prevented. In cases where prevention is not possible, agricultural insurance will be provided through the insurance pool infrastructure managed by private insurance companies and agencies, and the insurance premiums will be covered by state guarantees.*” (The AB Party)^83^  “*Our party will establish a holistic management network for our water resources, integrating them with evolving software technologies to create a system where they complement and support each other. Waters returning from agriculture, flowing rivers, and groundwater, along with rainfall cycles, will be addressed through an integrated strategy and action plan based on scientific research. We will effectively combat floods, inundations, and droughts*.” (The AA)^79^  “*Production and supply conditions should be established to enable the resumption of agricultural production in the disaster-affected areas.*” (The USF)^84^  “*Measures to increase productivity and enhance agriculture, such as soil reclamation, erosion prevention, opening new agricultural areas, revitalizing mountains with olive and fruit trees, terracing, constructing irrigation canals and dams, improving livestock, modernizing fisheries, and protecting forests and nature, will be achieved through cooperation and mutual efforts. Necessary machinery, equipment, tractors, agricultural chemicals, fertilizers, feed, seeds, and credit will be provided, especially to production cooperatives, to support these efforts.*” (The VP)^80^ |
|  | | Productivity and diversity in production | “*We will determine the crop patterns and plan production by considering the land's productivity potential as well as rainfall and irrigation capabilities.*” (The PA)^72^  “*We will develop seed gene banks and ensure the preservation and use of heritage and local seeds*.” (The NA)^71^  “*We will increase product and market diversification in poultry product exports*.” (The MP^a^)^42^  “*We will focus on natural seeds and establish 'Seed Banks' to pass these seeds on to future generations. We will also ban the import of seeds from foreign countries*.” (The AA)^75^  “*The seed legislation that benefits companies should be revised*.” (The USF)^85^  “Considering the biodiversity of regions, the cultivation of different types of plants in these areas will be encouraged in a rational manner.” (The MİLLİYOL)^86^  “*Emphasis will be placed on research and development (R&D) efforts, and government incentives will be provided unconditionally for the advancement of aquatic products*.” (The AB Party)^83^  “*We will not permit the use of GMO and hybrid seeds, and we will encourage the use of heritage seeds*.” (The LFA)^73^  “*We will encourage livestock fattening and establish breeding farms for efficient breeds*.” (The YP)^87^  “*To strengthen our agriculture sector and increase productivity, we will modernize our irrigation systems. We will teach farmers the latest agricultural technologies and organize training programs in this area. We will also support rural agricultural businesses with digital technologies to enhance their productivity.*” ( The BTP^a^)^76^  “*To successfully implement measures that will increase productivity and develop agriculture, such as soil reclamation, erosion prevention, opening new agricultural areas, revitalizing mountains with olive and fruit trees, terracing, constructing irrigation canals and dams, improving livestock, modernizing fisheries, and protecting forests and nature, necessary machinery, equipment, tractors, agricultural chemicals, fertilizers, feed, seeds, and credit will be provided, especially to production cooperatives, through cooperative and mutual efforts*.” (The VP)^80^ |
|  | | Food Waste and Loss | “*For the nutrition of large cities, we will support small family businesses on the city outskirts and develop a cold chain infrastructure to reduce transportation costs for agricultural products coming from neighboring provinces and prevent losses during the transport process*.” (The MP^a^)^42^  “*To prevent food waste from farm to table, we will promote the use of artificial intelligence and digital technology. We will support non-profit organizations that manage surplus or health-appropriate donated foods, preserving them under suitable conditions and delivering them directly or through institutions to those in need. We will also ensure that unusable foods are used in organic fertilizer production. Additionally, we will implement regulations to facilitate discounts on products nearing their expiration dates in markets*.” (The NA)^40^  “*We place great importance on specializing in agricultural production based on the characteristics of different regions of our country, and on developing support systems and agricultural management systems within this framework. While increasing access to food, we also prioritize policies aimed at preventing food loss and waste*.” (The PA)^72^  “*In the agricultural-food system, we will promote circular economy practices, such as converting market waste into compost, to reduce vegetable and fruit losses during transportation and retail processes, with the participation of municipalities and producer organizations*.” (The LFA)^77^  “*We must find ways to prevent waste and create efficiency in the use of energy, water, and food. This will slow down the depletion of our critical resources during crucial times. Technology will guide us in the process of increasing efficiency. In this context, educating the community is also important for achieving our goals*.” (The AA)^75^  “*By supporting ecological farming practices, we will develop projects to reduce and recycle food waste*.” ( The BTP^a^)^76^ |
|  | | Environmental protection and climate change | “*We will transform the Bahri Dağdaş International Agricultural Research Institute in Konya into a center for climate change adaptation, serving as the Bahri Dağdaş Climate Change Adaptation Center*.” (The NA)^71^  “*In addition to our geothermal resources, we will rapidly expand Agriculture-Based Specialized Organized Industrial Zones that utilize renewable energy sources such as wind, solar, and biomass.*” (The PA)^72^  “*In the long term, we will establish agricultural research and application centers that will reduce the need for petroleum-based external inputs, preserve soil fertility and resilience, provide resistance to climate change, and protect public health by researching and promoting both traditional and modern 'agroecological' practices*.” (The LFA)^77^  “*In the face of the food system controlled by corporations, the only system that is dignified for people and harmonious with nature—public food sovereignty—must be implemented*.” (The USF)^85^  “*We will implement the 'Climate Change and Drought Action Plan' without delay. We will support innovative research in this field, starting with the development of drought-resistant seeds*.” (The MP^a^)^42^  “*We will support organic farming*.” (The YP)^87^ |
|  | Institutional and systemic changes (category) |  | “*We will manage Marine and Inland Fishery Stocks and Reserves in accordance with the National Master Plan*.” (The NA)^71^  “*We will establish a National Rural Network to manage ongoing projects in agriculture and rural development under a single umbrella of different institutions and organizations*.” (The PA)^72^  “*We will utilize digital technologies to conduct a comprehensive agricultural census in areas such as farmers, land use, climate, irrigation, crop patterns, and agricultural production profiles, creating the 'Türkiye Farmer and Agriculture Inventory'*.” (The MP^a^)^42^  "*All privatized public institutions should be restructured in a farmer-friendly and democratic manner to promote and encourage ecological peasant agriculture*." (The USF)^85^  “*We will establish a cooperative bank and an Agricultural Development Fund to support agricultural cooperatives*.” (The AA)^75^  “*We will shape agricultural policy with a dynamic and holistic perspective, addressing environmental, social, and economic benefits together, defining tools and policies suitable for different regions and periods, and evaluating their impact. To facilitate this planning, we will establish a public information system that collects and provides accessible input-output and cost information for all goods, products, and services in the agriculture-food system*.”  (The LFA)^77^  “*The demands of organizations such as the European Union, IMF, and World Trade Organization will be rejected, and agriculture will be supported*.” (The VP)^80^  “*We will establish Provincial Agricultural Authorities in every province*.” (The YP)^87^  “*The registration of modern and active (bee-containing) beehives in the Livestock Information System (HAYBİS) and the Beekeeping Record System (AKS) will be mandatory. These beehives will be insured against damages resulting from natural disasters such as storms, tornadoes, fires, landslides, earthquakes, vehicle collisions, floods, wild animal attacks, and accidents during transportation, such as collisions, impacts, tipping, or burning (applicable only for migratory beekeeping). In cases where damages cannot be prevented, insurance will be provided through special insurance companies and agencies using the TARSIM (Agricultural Insurance Pool) infrastructure, with the premiums covered by state guarantees. Additionally, the importance of R&D efforts and the development of beekeeping will be supported by providing unconditional state incentives*.” (The AP Party)^83^  “*We will establish an R&D center in each provincial and district agriculture directorate. In the shortest time possible, we will allocate agricultural district unions and provincial agricultural unions that collaborate with, guide, and assist farmers*.” (The MİLLİYOL)^86^  “*We will provide young farmers with opportunities to enhance their knowledge and skills through agricultural training and career development projects*.” (The BTP^a^)^76^  “Agriculture needs to be equipped with the latest agricultural tools and production methods.” (The HKP)^88^ |
| Food Safety | |  | “*We will introduce quality standards in the honey sector and carry out the necessary inspections for counterfeit and adulterated products*.” (The NA)^71^  “*In the prevention of animal diseases, the expert opinions of veterinarians will be prioritized. Veterinary services' provincial organizations will be established within the Ministry of Food, Agriculture, and Livestock, ensuring an effective fight against animal diseases through this approach*.” (The PA)^89^  “*We will ban the use of pesticides, chemical fertilizers, and GMOs in agriculture*.” (The LFA)^73^  “*In terms of food safety, we will monitor and inspect agricultural products at every stage, from farm to table, in the most effective way*.” (The MP^a^)^42^  “*While encouraging the use of local seeds, we will end the practice of imported seeds. We will lift the bans on local seeds. We will prevent the use of genetically modified products and seeds in food production and imports*.” (The AA)^75^  “*Agriculture and livestock should be restructured based on public ownership, ending external dependency. Türkiye must provide affordable, quality, and healthy food to all its citizens*.” (The USF)^74^  “*Businesses with disease-free certification and biosecurity measures in place will be supported. Unconditional state incentives will be provided to prioritize R&D efforts and promote cattle farming*.” (The AB Party)^83^  “*We will impose severe penalties on food producers who endanger public health*.” (The YP)^87^  “*Veterinary faculties will be transformed into laboratory fields. Facilities such as farm analysis centers and feed factories will be established*.” (The MİLLİYOL)^86^  “*Citizens who cultivate the soil and produce value from it will receive advances even before planting the seeds. With the support of agricultural engineers and veterinarians working within municipal frameworks, production quality will be improved. First, we will assist in feeding our people with quality and healthy food*.” (The BTP^a^)^76^ |
| Food Supply | |  | “*To ensure supply security, sustainability, and predictability in production, we will implement a model that guarantees the income of raw milk and red meat producers.*” (The PA)^72^  “*Within the Ministry of Agriculture and Food, we will establish expert panels for selected strategic products. These panels will monitor domestic prices, production, supply, demand, stock balance, as well as global production, stock, and price developments on a daily basis. They will implement timely active foreign trade policies as needed and strengthen policies related to supply security*.” (The NA)^43^  “*With the policies we will implement, we aim to establish an agricultural sector that focuses on sufficient and balanced nutrition for society, is based on advanced technology, information, and data, resolves infrastructure issues, has high organization and efficiency, practices planned production with supply-demand balance, enhances international competitiveness, and uses natural resources (soil, water basins, water sources) in a sustainable manner*.” (The MP^a^)^42^  “*Food prices should be frozen, and the daily needs of the public should not be left to the whims of market profiteers and opportunists. All supermarkets and food supplies should be consolidated under a single distribution cooperative.*” (The USF)^91^  “*We will aim for self-sufficiency in food production, medical supplies and pharmaceutical industries*.” (The LFA)^77^  “*Sustainability in production is ensured by securing product supply. To address this, the Victory Party will revisit the base price policy for all products to ensure supply security, both in terms of raw materials needed by the industry and consumer demand. Contract farming will be integrated within the agricultural sector, addressing all aspects, to prevent the crisis of farmers having to wait for buyers in the field or experiencing production disruptions due to lack of supply guarantees*.” (The AA)^79^  “*We will deliver agricultural and animal products obtained from municipal farms to our citizens through municipal markets that we will establish in each neighborhood, operated by the municipality itself. Only residents of our city or district will benefit from these markets. As intermediaries and distances will be eliminated, and because it will be a non-profit, large-scale production, prices will drop to half of today's sticker prices. By supporting agriculture in our villages and cities, we will ensure food production within our own borders and eliminate depende*ncy.” (The BTP^a^)^76^  “*We will make Türkiye the largest supply center for food production and agriculture in the region and the world. To achieve this, we will implement a new administrative structure by establishing Provincial Agricultural Authorities in each province*.” (The YP)^87^ |
| Cultural Sensitivity | |  | “*Considering that the majority of the population is Muslim, regulations regarding halal food and halal slaughter should be established*.” (The PA)^45^  “*We will create flavor and tasting routes in our country and establish gastronomic streets in our tourist regions*.” (The NA)^71^ |

*: The content or themes mentioned in the table are provided as examples for each party/ alliance. It is given one pledge for each alliance/non-aligned party. Parties and alliances not listed in the table did not have a pledge related to this code or category. The examples provided for each code or category are put in descending order from the party or alliance that produced the greatest number of pledges to the least.

**Supplementary Table 6:** Pledges given by parties/alliances in related Sustainable Development Goals*.

| Themes | Quotes |
| --- | --- |
| Goal 2: Zero Hunger | “*We will ensure food safety and food security from farm to table.”* (The NA)^71^  “*Our policies will focus on establishing food supply security through a robust agricultural production system tailored to the needs of our population. Ensuring continuous physical and economic access to adequate, healthy, and reliable food will be a core element of our approach*.” (The PA)^72^  “*We will exhaust all resources to ensure access to basic food at affordable prices*.” (The LFA)^91^    “*Food bank distributes donated or surplus food items to those in need. Leading firms in food distribution and production in Turkey support these food banks as part of their corporate responsibility and benefit from tax advantages. We will provide incentives to promote the expansion of this concrete initiative based on the win-win principle. Similar to the EU, we will exempt donated food from VAT*.” (The MP^a^)^42^  “*State-run public bread factories and soup kitchens should be established immediately in every district and provide free services. Public cooperatives should be opened in every neighborhood. The public should not be subjected to a monopoly of just five markets. Water, bread, and milk for children should be distributed for free!*” (The USF)^92^    “*Distributed production principles will apply to all aspects of our lives. We will produce energy, water, and food and consume what we produce within our own region or nearby*.” (The AA)^75^  “*We will put an end to price terrorism in food*.” (The YP)^87^  “*Goods produced for the food, clothing, and heating needs of low-income individuals will be delivered to the public through a chain of retail stores.”* (The VP)^80^  “*Dairy and male beef cattle, as well as buffaloes, will be required to be registered in the Livestock Information System (HAYBİS). For these large livestock, insurance will be provided for damages due to various conditions, including: animal diseases, pregnancy, childbirth or surgical interventions, accidents, wild animal attacks, snake and insect bites, poisoning from toxic pasture plants and feed, natural disasters, sunstroke, fires or explosions. In cases where prevention is not possible, insurance will be arranged through specialized insurance companies and agencies using the TARSİM infrastructure, and the insurance premiums will be covered by state guarantees.*” (The AB Party)^83^  “*Farmers who work the land and produce value from it will be provided with advances before planting seeds. The quality of production will be improved with the support of agronomists and veterinarians working within the municipality. Our priority will be to help feed our people with high-quality and healthy food*.” (The BTP^a^)^76^    “*Animal husbandry will be transitioned from traditional methods to a modern framework in light of technological advancements. Projects will be developed based on increasing efficiency and production*.” (The MİLLİYOL)^86^  “*In agriculture, the first step we must take is the immediate conversion of unused state and private lands into agricultural use through a genuine land reform. The second step is to equip agriculture with the latest agricultural tools and production methods.*” (The HKP)^88^ |
| Goal 5: Gender Equality | “*We will apply the principle of positive discrimination for seasonal female agricultural workers and ensure that local governments take responsibility.*” (The LFA)^73^  “*We will provide incentives to ensure that women have a greater role in both subsistence farming and in new agriculture based on productivity and innovation*.” (The NA)^71^  “*We will provide additional support to young people and women. We will also assist with social security premiums for young and female farmers*.” (The PA)^72^  “*We will develop policies to support women, female producers, female entrepreneurs, and young people in agricultural production. We will include young people employed in agriculture in the social security system and ensure that there are no uninsured agricultural workers*.” (The MP^a^)^42^  “*Legislation that obstructs rural women’s access to credit, education, and all forms of universal rights should be changed*.” (The USF)^85^ |
| Goal 6: Clean Water and Sanitation | “*We will ensure the establishment of 'Wastewater Treatment Plants' in all settlement areas and will not grant credit or approval for wastewater channel projects without a treatment facility*.” (The NA)^71^  “*We will plan to place water storage tanks and reservoirs, similar to cistern culture, under green areas, connected to the city water supply for clean drinking water usage. These systems will be designed to automatically seal themselves in case of an emergency.*” (The PA)^72^  “*We will guarantee the right to access clean water for everyone*." (The LFA)^77^ |
| Goal 12: Responsible Consumption and Production | “*We will increase awareness through public service announcements to reduce household food waste. This includes avoiding the purchase of more food than needed, not buying excessive amounts of food that will not be used immediately, minimizing uneaten food, and avoiding processes that lead to a loss of food quality*.” (The MP^a^)^42^  “*We will prevent food waste by identifying loss points from farm to fork in agricultural and processed food products*.” (The NA)^71^  “*We will promote Urban Agriculture practices to shorten the supply chain from farm to table, reduce logistics costs, maintain product freshness, protect the environment by reducing carbon emissions, and increase local employment through the concept of local production-local consumption.*”(The PA)^72^  “In the agricultural-food system, we will reduce fruit and vegetable losses in the transportation and retail process by promoting circular economy practices (such as composting market waste) with the participation of municipalities and producer organizations.” (The LFA)^77^  “*We must find ways to prevent waste and create efficiency in the use of energy, water, and food. This will slow down the depletion of our vital resources during critical times. Technology will guide us in increasing efficiency, and educating the public will be crucial in achieving these goals*.” (The AA)^75^  “*We will support ecological farming practices and develop projects to reduce food waste and promote recycling*.” (The BTP^a^)^76^ |

*: The content or themes mentioned in the table are provided as examples for each party/ alliance. It is given one pledge for each alliance/non-aligned party. Parties and alliances not listed in the table did not have a pledge related to this code or category. The examples provided for each code or category are put in descending order from the party or alliance that produced the greatest number of pledges to the least.
